# Taking down ‘the Ivory Tower’: leveraging academia for better health outcomes in Uganda

**DOI:** 10.1186/1472-698X-11-S1-S1

**Published:** 2011-03-09

**Authors:** Joseph C Kolars

**Affiliations:** 1 Professor of Medicine, Senior Associate Dean of Education and Global Initiatives, University of Michigan Medical School, Ann Arbor, MI 48109-5624, USA

## 

***ivory tower,*** noun (1) *an impractical often escapist attitude marked by aloof lack of concern with or interest in practical matters or urgent problems; (2) a secluded place that affords the means of treating practical issues with an impractical often escapist attitude;* especially *a place of learning*[1]

Sub-Saharan Africa (SSA) is home to less than ten percent of the world’s population but bears nearly a quarter of the world’s disease burden. At the same time, the region has limited financial resources to invest in solutions [2]. The international community has commonly responded with narrowly focused, short term, technical approaches or relatively simplistic aid programs. The absence of a comprehensive strategy or “systems approach” has resulted in a patchwork of well-intended but often poorly coordinated initiatives. While academic institutions in the North (i.e. North America and Europe) have sought opportunities to be helpful, concerns have been raised regarding the focus of their initiatives and misalignments with the priorities of host countries. Funding for such initiatives typically originate outside of the region and while academic institutions in the North have clearly benefited from these opportunities, the value to the host institutions or to health systems in SSA is often much more difficult to determine.

Another concern pertains to the relevance of academic institutions in the face of dire global health challenges. What role can academic institutions play in creating practical solutions to tackle pressing societal problems? By their nature, institutions of higher learning are concerned with the creation of new knowledge, while often leaving the application of their findings to others. They rarely evaluate the relevancy of their training programs to the current needs of the health care system. Implementation has not traditionally been considered as part of the academic agenda. In addition, stakeholders who are most familiar with the problems that hamper improvements in health are usually not members of academic communities. As a result, civil society and government agencies, such as the Ministries of Education and Health, often view academicians as intellectuals who are cloistered in their Ivory Towers, seemingly uninterested or unwilling to deal with the pressing problems of the day. In the case of global health, an ever growing number of non-governmental organizations have stepped forward to work on solutions, with academic institutions watching from the sidelines.

This collection of papers, focused on work conducted at Makerere University College of Health Sciences (MakCHS) in Uganda, challenges the Ivory Tower notion with a set of approaches and findings that are a testimony to the relevance of a leading academic institution to the health problems of SSA. The papers range from assessments of the educational approaches and ability to provide health services, to policy and strategic analysis needed for sustainability of the institution, to research projects that demonstrate the practical role of the University in the health sector. Furthermore they demonstrate a new model of collaboration between universities in low- and high-income countries; a model that emphasizes local leadership and ownership.

Most of the papers in this supplement are based on work that was funded by the Bill and Melinda Gates Foundation (BMGF). With the belief that health science schools have a critical role in improving health care in SSA, the BMGF initiated their Collaborative Learning Initiative in 2008 with two-year learning grants designed to demonstrate and incentivize the accountability of health professions schools to the problems of society [3]. These grants brought together leading health science schools in SSA with partner schools in the U.S. to explore the value proposition that academia could bring to the pressing health problems in the region. To be sure, these relationships have existed for many years, long preceding the initiation of these learning grants. However, this initiative was unique in challenging these collaborations to focus on generating evidence that the academic solutions are relevant to the needs of local populations.

The initiative focused on three dimensions of collaborative partnerships. First, the high-level strategic planning and coordination between schools and Ministries of Health, which is often missing in SSA, was deemed essential to these initiatives. Second, the collaborations were to extend beyond schools of medicine, where the power and influence has often been centered, to the exclusion of other key disciplines. Multi-disciplinary approaches involving nursing, public health, and other health professionals were sought. Third, the collaborations between the academic institutions in SSA and the North were to be centered on the needs of the African countries, rather than the research needs of the North. What follows is a remarkable collection of papers reflecting the evidence brought forth by a collaboration between MakCHS and Johns Hopkins University (JHU) in the United States. To many, these are two of the tallest and brightest of our Ivory Towers, each with a long track-record of ground-breaking discoveries. Makerere University, founded in 1922, is one of the oldest and most prestigious universities in Africa. Each year they graduate 600 health professionals from 23 degree programs, many of whom take on leadership positions that influence healthcare in Uganda and across the region. A vibrant research program has created partnerships with a number of international research institutions worldwide. Johns Hopkins University is one of the top research institutions in the world with strong schools of medicine, public health, and nursing. They successfully compete for global health funding and have secured a number of grants and contracts in partnership with MakCHS from entities such as the National Institutes of Health and the Presidents Emergency Plan for AIDS Relief (PEPFAR) in the United States, private-public partnerships such as the Global Fund for AIDS, Tuberculosis and Malaria, international agencies, and foundations. In 2009, MakCHS and JHU initiated four pilot projects to examine the ability of academic institutions to work meaningfully on the most challenging health problems in Uganda. They also set out to analyze the value of past collaborations and initiatives to the local population.

Collectively, these reports address three lines of evidence regarding the benefits of academic partnerships to the health problems of Uganda. First, research from MakCHS has had a transformative impact on health policy in Uganda, and examples from efforts to reduce HIV transmission are explored in detail. An analysis of the 837 papers published by MakCHS faculty between 2005-9 demonstrates good alignment with the priorities of the Ugandan Health Ministry. Second, academic partnerships with local communities have strengthened care. The Safe Deliveries Pilot Project with two districts in Eastern Uganda has brought a diverse set of stakeholders together on an innovative approach that utilizes local resources to improve maternal and neonatal care. Partnerships with government clinics in Kampala have resulted in multi-disciplinary approaches to improving the health of disadvantaged urban populations. Makerere University College of Health Sciences has made a major commitment to Community-Based Education and Services (COBES) for health professionals in under-served rural areas. This includes service learning programs for students that are designed to improve the placement of healthcare workers in these areas of need.

The third line of evidence regarding the value proposition of academic partnerships pertains to the efforts undertaken by MakCHS to ensure that its infrastructure, programs, and processes are optimized to address the health needs of Uganda. With respect to education, the curricula focuses on essential competencies and what healthcare workers are able “to do” rather than previous paradigms that limit themselves to what trainees are expected “to know” [4]. These competencies are in close alignment with the Ugandan government’s Health Sector Strategic Plan. As with most education systems world-wide, MakCHS is struggling to improve its ability to ensure that the desired competencies are in fact present in their graduates, but this will be the focus of future efforts. MakCHS has also recognized opportunities for improvements in the teaching of professionalism and communication skills, as well as processes for quality improvement. The development of a more robust grants management office will increase opportunities for taking the lead on funding proposals coming forth from the international community. The ability of MakCHS and JHU to critically reflect on their collaborative efforts and take stock of their progress to date, as demonstrated in these papers, is predictive of further changes and improvements.

It is clear that MakCHS has defined the work they have been doing on health systems as core to their academic mission. They have also demonstrated the value of this work to the population that they serve. The health system needs competent health care workers who are appropriately distributed and have the resources and processes in place to ensure effectiveness. The research questions should be centered on the needs of Uganda, and they should emanate from stakeholders that are likely to reside beyond the realm of academia. Partnerships, such as the one with JHU reflected in these reports, represent an approach that is likely to lead to strengthening of institutions in SSA.

The research agenda required for academic institutions to bring improvements to the healthcare system in SSA is substantial. By definition, multi-disciplinary efforts and cross-institutional efforts will be required. The recent Sub-Saharan African Medical School Study documents the poor state of medical education in the region as well as the benefits to collaborative approaches and sharing of best practices [5]. However, none of this can take place in the absence of adequate funding. Funding initiatives that bypass academic institutions because of a reluctance to fund Ivory Tower initiatives need to be reexamined. Recent initiatives, such as the Medical Education Partnership Initiative from PEPFAR and the National Institutes of Health will invest approximately $130 Million USD over the next five years to strengthen Africa’s educational institutions to produce the quantity and quality of scientists and healthcare workers needed to address the healthcare problems in the region [6]. Whereas this represents a step in the right direction, substantially more funding will be required, including funding from the African governments themselves, to address national health priorities.

In summary, this body of work represents the power of collaboration and challenges conventional notions that academia is hesitant to come down from their ivory towers. Universities can and must be socially relevant. Funding and investments are needed now to make these collaborations sustainable.

## List of abbreviations used

BMGF: Bill and Melinda Gates Foundation; COBES: Community-Based Education and Services; JHU: Johns Hopkins University; MakCHS: Makerere University College of Health Sciences; PEPFAR: (United States) Presidents Emergency Plan for AIDS Relief; SSA: Sub-Saharan Africa.

## Competing interests

Dr. Kolars serves as a Consultant for the Bill and Melinda Gates Foundation.

## References

[B1] Merriam Webster Dictionary “ivory tower” http://www.merriam-webster.com/dictionary/ivory%20tower

[B2] World Health Organization Working Together for Health: The World Health Report 2006 2006 Geneva, Switzerland: WHO Press

[B3] Kolars JC Cahill K Donkor P Kaaya E Lawson A Serwadda D Sewankambo NK Partnering for medical education in Sub-Saharan Africa – seeking the evidence for effective collaborations Academic Medicineunder review 10.1097/ACM.0b013e31823ede39 22189887

[B4] Frenk J Chen L Bhutta ZA Cohen J Crisp N Evans T Fineberg H Garcia P Ke Y Kelly P Kistnasamy B Meleis A Naylor D Pablos-Mendez A Reddy S Scrimshaw S Supulveda J Serwadda D Zurayk H Health professionals for a new century: transforming education to strengthen health systems in an interdependent world Lancet 2010 376 1923 1958 10.1016/S0140-6736(10)61854-5 21112623

[B5] Mullan F Frehywot S Omaswa F Buch E Chen C Greysen SR Wassermann T Abubakr DE Awases M Boelen C Diomande MJ Dovlo D Ferro J Haileamlak A Iputo J Jacobs M Koumaré AK Mipando M Monekosso GL Olapade-Olaopa EO Rugarabamu P Sewankambo NK Ross HR Ayas H Chale SB Cyprien S Cohen J Haile-Mariam T Hamburger E Jolley L Kolars JC Kombe G Andre-Jacques AJ Neusy Medical schools in sub-Saharan Africa Lancet 2010 10 1961 1967 10.1016/S0140-6736(10)61961-7 21074256

[B6] Collins FS Glass RI Whitescarver J Wakefield M Goosby EP Developing health workforce capacity in Africa Science 2010 330 1324 1325 10.1126/science.1199930 21127233 PMC5101931

